# miR-34a-Mediated Survivin Inhibition Improves the Antitumor Activity of Selinexor in Triple-Negative Breast Cancer

**DOI:** 10.3390/ph14060523

**Published:** 2021-05-29

**Authors:** Silvia Martini, Valentina Zuco, Monica Tortoreto, Stefano Percio, Elisa Campi, Rihan El Bezawy, Valentina Doldi, Yosef Landesman, Marzia Pennati, Nadia Zaffaroni

**Affiliations:** 1 Molecular Pharmacology Unit, Department of Applied Research and Technological Development, Fondazione IRCCS Istituto Nazionale dei Tumori, 20133 Milano, Italy; silvia.martini@istitutotumori.mi.it (S.M.); valentina.zuco@istitutotumori.mi.it (V.Z.); monica.tortoreto@istitutotumori.mi.it (M.T.); stefano.percio@istitutotumori.mi.it (S.P.); elisa.campi@istitutotumori.mi.it (E.C.); rihan.elbezawy@istitutotumori.mi.it (R.E.B.); valentina.doldi@istitutotumori.mi.it (V.D.); marzia_pennati@hotmail.com (M.P.); 2 Karyopharm Therapeutics, Newton, MA 02459, USA; ylandesman@karyopharm.com

**Keywords:** negative breast cancer, selinexor, miR-34a, survivin, xenografts, apoptosis

## Abstract

Triple-negative breast cancer (TNBC) is an aggressive disease with limited therapeutic options. Here, we pursued a combinatorial therapeutic approach to enhance the activity of selinexor, the first-in-class XPO1 inhibitor, by miR-34a ectopic expression in human TNBC experimental models. Anti-proliferative activity induced by selinexor and miR-34a expression, singly and in combination, was evaluated by MTS assay and cell counting. The effect of treatments on survivin and apoptosis-related proteins was assessed by western blotting and ELISA. The antitumor and toxic effects of individual and combined treatments were evaluated on TNBC orthotopic xenografts in SCID mice. Selinexor consistently showed anti-proliferative activity, although to a variable extent, in the different TNBC cell lines and caused the impairment of survivin expression and intracellular distribution, accompanied by apoptosis induction. Consistent with in vitro data, the XPO1 inhibitor variably affected the growth of TNBC orthotopic xenografts. miR-34a cooperated with selinexor to reduce survivin expression and improved its anti-proliferative activity in TNBC cells. Most importantly, miR-34a expression markedly enhanced selinexor antitumor activity in the less sensitive TNBC xenograft model, in absence of toxicity. Our data form a solid foundation for promoting the use of a miR-34a-based approach to improve the therapeutic efficacy of selinexor in TNBC patients.

## 1. Introduction

Triple-negative breast cancer (TNBC) is an aggressive form of breast cancer (BC), defined by the lack of estrogen receptor (ER), progesterone receptor (PR), and human epidermal growth factor receptor 2 (HER2), which accounts for 15–20% of all BC cases [1]. Although initially chemotherapy sensitive, TNBC displays high rates of local recurrence and distant metastasis [2]. Metastatic TNBC is associated with a poor prognosis and the development of novel therapeutics represents a major unmet clinical need.

Based on the notion that altered localization of proteins, which highly affects their biological functions, is a common feature in cancer [3], the regulation of protein trafficking between the nucleus and cytoplasm has been recently regarded as a novel control point for therapeutic interventions [4,5]. In this context, exportin 1 (XPO1), the sole nuclear exporter for a variety of tumor suppressors, cell cycle regulators and growth promoting/anti-apoptotic proteins [6], was found to be over-expressed in several cancer types, including BC [7].

Selective inhibition of XPO1 by selinexor, a small molecule approved for the treatment of relapsed or refractory multiple myeloma and relapsed/refractory diffuse large B-cell lymphoma and currently under clinical development in a variety of hematological and solid tumors [8], has been shown to induce anti-proliferative and anti-tumor activity in experimental models of different tumor types [4], including TNBC cell lines and xenograft models [9,10,11]. However, results from a Phase II trial indicated that, although generally well tolerated, single-agent selinexor has limited activity in heavily pretreated patients with metastatic TNBC [12], suggesting the opportunity to focus on combination regimens aimed at increasing its therapeutic effect. 

We and other previously demonstrated that down-regulation of the anti-apoptotic protein survivin is an important determinant of the apoptotic response through which selinexor induces its anti-tumor activity in experimental models of different tumor types [13,14,15,16]. Looking for a strategy aimed at improving the activity of selinexor in TNBC experimental models, in this study we investigated the effect exerted by the ectopic expression of miR-34a, an onco-suppressive miRNA known to be down-regulated in different tumor types [17], including TNBC [18], and previously reported to target survivin [19,20,21], on the in vitro and in vivo activity profile of selinexor. Results are reported herein.

## 2. Results

### 2.1. Selinexor Inhibits TNBC Cell Growth 

**Figure 1:**
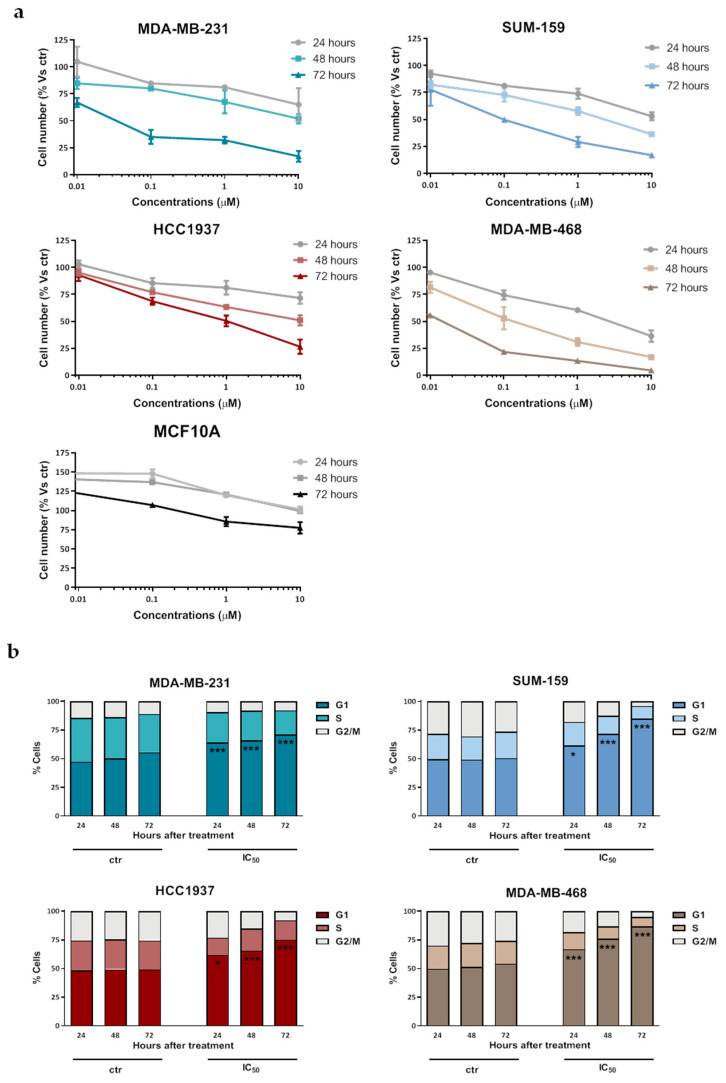
Effect of selinexor on cell growth and cell cycle distribution of TNBC cell lines. (**a**) MDA-MB-231, SUM-159, HCC1037, MDA-MB-468 cells and human normal breast cells (MCF-10A) were cultured for different time intervals in the presence of increasing concentrations of selinexor or 0.1% (*v*/*v*) DMSO (control cells; ctr), and the cytotoxic activity was assessed by MTS assay. Data are expressed as the percentage of cells (mean values ± SD of three independent experiments) in each conditions with respect to control cells (ctr). (**b**) Flow cytometric analysis of cell cycle distribution in TNBC cells exposed to 0.1% (*v*/*v*) DMSO (control cells; ctr) and selinexor (IC_50_ at 72 h, as reported in Table 1). Data are reported as the percentage of cells in G1, S, and G2/M phases and represent the mean values of three independent experiments; SDs were always within 5%. Only G1 phase comparisons were explicited. * *p* < 0.05, *** *p* < 0.005 Tukey’s *post hoc* test.

Selinexor cytotoxic activity was assessed by MTS assay in a panel of human TNBC cell lines (MDA-MB-231, SUM-159, HCC1937 and MDA-MB-468). The drug consistently induced a dose- and time-dependent inhibitory effect on cell growth (Figure 1a), although cell lines showed a huge variability in their sensitivity to the compound, as indicated by the IC_50_ values at different exposure times (Table 1). Conversely, selinexor did not appreciably inhibit the growth of the human normal breast cell line MCF10A (Figure 1a).

Consistent with the XPO1 role as nuclear export mediator of several cell cycle regulatory proteins [6], selinexor induced a time-dependent G1 phase accumulation in all TNBC cell lines, as detected by flow cytometry after 72 h exposure to an equally effective dose (IC_50_ at 72 h) drug concentration (Figure 1b). 

### 2.2. Selinexor Impairs Survivin Expression and Intracellular Distribution, and Enhances Apoptosis in TNBC Cells

Based on previous evidence showing an important role of the anti-apoptotic protein survivin in mediating selinexor activity in experimental models of different tumor types [10,13,14,15,16], we assessed the expression and subcellular distribution of the protein. Indeed, survivin distribution is regulated by active import into the nucleus and XPO1-mediated export to the cytoplasm [22], and the anti-apoptotic activity of the protein is associated with its cytoplasmic/mitochondrial pool [23].

**Figure 2:**
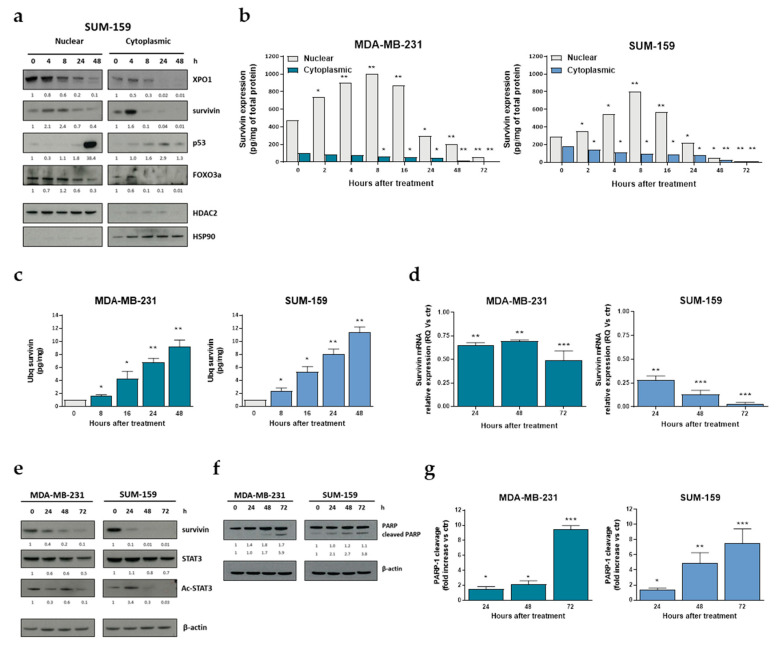
Effect of selinexor on survivin expression in TNBC cell lines. (**a**) Representative western blot showing nuclear and cytoplasmic fractions of XPO1 and survivin in TNBC cells exposed to selinexor (IC_50_ at 72 h, as reported in Table 1). HDAC2 and HSP90 were used to confirm equal protein loading on the gel and to show the relative purity of the nuclear and cytosolic fractions, respectively. Cropped images of selected proteins are shown. For each protein, band density was quantified using ImageJ normalized to loading control and referred to respective untreated control.(**b**) Quantification of nuclear and cytosolic survivin protein levels by ELISA assay in TNBC cells exposed to selinexor (IC_50_ at 72 h). Data are reported as amount (pg) of survivin normalized to total (mg) protein extract, and represent the mean values ± SD of three independent experiments. (**c**) Quantification of ubiquitinated (Ubq)-nuclear survivin protein levels by ELISA assay in TNBC cells exposed to selinexor (IC_50_ at 72 h). Data are reported as amount (pg) of Ubq-nuclear survivin normalized to total (mg) nuclear protein extract, and represent the mean values ± SD of three independent experiments. (**d**) Quantification of survivin mRNA expression levels by qRT-PCR in TNBC cells exposed to selinexor (IC_50_ at 72 h) at different intervals. Data are reported as relative quantity (RQ) in selinexor-treated cells with respect to control cells exposed to 0.01% DMSO (ctr), and represent the mean values ± SD of three independent experiments. (**e**) Representative western blotting showing the expression of survivin, STAT3 and Ac-STAT3 in TNBC cells exposed to selinexor (IC_50_ at 72 h) at different intervals. β-actin was used to confirm equal protein loading on the gel. Cropped images of selected proteins are shown. (**f**) Representative western blotting showing the cleavage of PARP-1 in selinexor-treated (IC_50_ at 72 h) TNBC cell lines. Cropped images of selected proteins are shown. (**g**) Quantification of PARP-1 cleavage. Data are expressed as mean values ± SD of three independent experiments. * *p* < 0.05, ** *p* < 0.01, *** *p* < 0.005, Student′s *t*-test.

Western blot results showed that in SUM-159 cells selinexor caused a nuclear accumulation of survivin appreciable until 8 h after treatment, which was followed by a progressive decrease in nuclear survivin content and paralleled by a time-dependent reduction of cytoplasmic survivin abundance (Figure 2a). Such results were then confirmed by ELISA in both MDA-MB-231 and SUM-159 cell lines (Figure 2b). 

According to previous findings indicating that forced retention of survivin in the nucleus promotes its clearance by the ubiquitin-proteasome proteolytic pathway [24], we found that exposure of TNBC cells to selinexor resulted in a time-dependent increase of ubiquitinated nuclear survivin, as assessed by immunoprecipitation and ELISA in both MDA-MB-231 and SUM-159 cell lines (Figure 2c). Selinexor also inhibited survivin gene transcription as indicated by the progressively reduced survivin mRNA abundance, particularly evident in SUM-159 cells (Figure 2d), which was paralleled by a time-dependent decrease in the acetylation levels of STAT3 in both cell lines (Figure 2e). This finding is in accordance with previous results obtained in the HER+ breast cancer cell line SKBR3 showing that the XPO1 antagonist KPT-276 repressed survivin transcription by inhibiting CBP-mediated STAT3 acetylation and blocking STAT3 binding to the survivin promoter [25].

Consistent with the anti-apoptotic role of survivin, selinexor-induced down-regulation of the protein was followed by a time-dependent apoptotic response, as detected by the cleavage of the PARP-1 by caspase-3 in both MDA-MB-231 and SUM-159 cell lines (Figure 2f,g). Nuclear accumulation of other XPO1 target proteins, such as p53 and Foxo3a, was observed after selienxor exosure of TNBC cells (Figure 2a).

### 2.3. Selinexor Variably Affects the Growth of TNBC Xenografts

**Figure 3:**
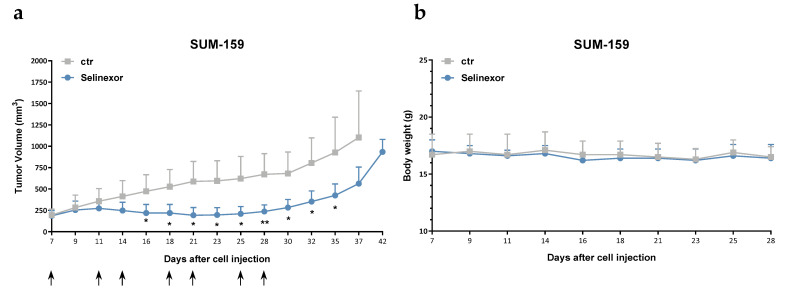
Efficacy of oral selinexor on TNBC xenografts. (**a**) Tumor growth curves of SUM-159 cells orthotopically injected into SCID mice. Mice were randomly grouped (6 mice/group) to receive vehicle (ctr) or selinexor (10 mg/kg, q3-4d × 8). Each point indicates the mean tumor volume in the group. Bars represent SD. Arrows indicate the day of the treatment. (**b**) Body weights of mice during the treatment period. Each point indicates the mean weight of mice in each group. Bars represent SD. * *p* < 0.05, ** *p* < 0.01, Student′s *t*-test.

The antitumor activity of oral selinexor was assessed in the TNBC cell models SUM-159 and MDA-MB-231, which are characterized by a different in vitro sensitivity to the compound (Table 1), following orthotopic xenotransplantation into SCID mice. Consistent with in vitro results, a remarkable antitumor effect was observed in SUM-159 xenografts, with a maximum tumor volume inhibition (TVI) of 67% (Figure 3a, Table 2). Conversely, selinexor treatment caused a negligible growth delay in MDA-MB-231 xenografts, as indicated by a maximum TVI of 25% (Table 2). The drug was well tolerated in both in vivo models, with no toxic deaths and minimal weight loss (Figure 3b). 

### 2.4. miR-34a Ectopic Expression Inhibits Survivin Expression and Induces TNBC Cell Growth Delay

**Figure 4:**
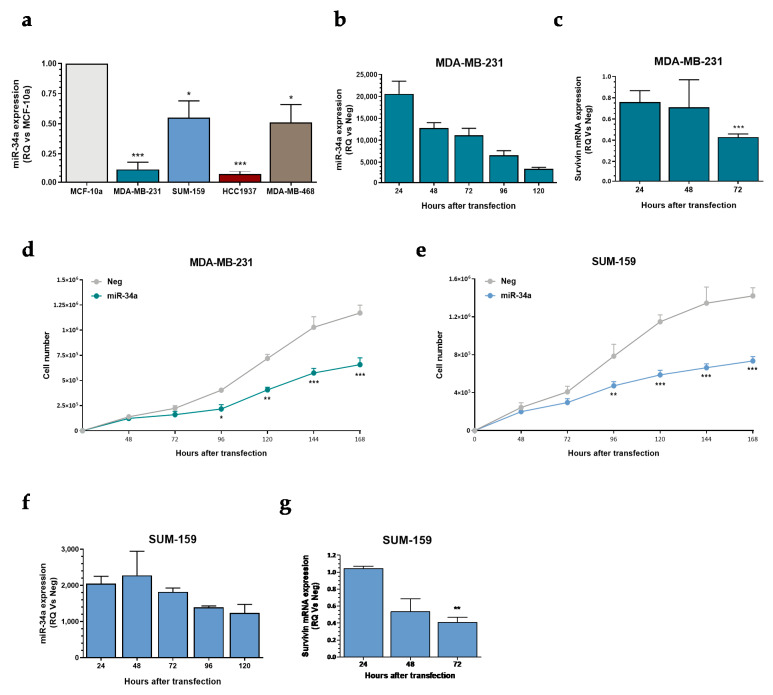
Effect of miR-34a ectopic expression on TNBC cells. (**a**) Quantification of basal miR-34a expression levels by qRT-PCR in TNBC cell lines. Data are reported as relative quantity (RQ) with respect to normal breast epithelial MCF10A cells, and represent mean values ± SD of three independent experiments. (**b**) qRT-PCR assessment of miR-34a expression levels in MDA-MB-231 cell line after transfection with the miRNA mimic. Data are reported as relative quantity (RQ) with respect to Neg-transfected cells, and represent mean values ± SD of three independent experiments. (**c**) Quantification of survivin mRNA expression levels by qRT-PCR in MDA-MB-231 cells transfected with Neg or miR-34a. Data are reported as relative quantity (RQ) in miR34a-transfected cells respect to negative controls (Neg), and represent the mean values ± SD of three independent experiments. (**d**) Effects of miR34a ectopic expression on MDA-MB-231 cell growth. Data are reported as number of growing cells over time, and represent mean values ± SD of three independent experiments. (**e**) Effects of miR34a ectopic expression on SUM-159 cells growth. Data are reported as number of growing cells over time, and represent mean values ± SD of three independent experiments. (**f**) qRT-PCR assessment of miR-34a expression levels in SUM-159 cell line after transfection with miR-34a mimic. Data are reported as relative quantity (RQ) with respect to Neg, and represent mean values ± SD of three independent experiments. (**g**) Quantification of survivin mRNA expression levels by qRT-PCR in SUM-159 cells transfected with Neg or miR-34a. Data are reported as relative quantity (RQ) in miR34a-transfected cells with respect to Neg, and represent the mean values ± SD of three independent experiments. * *p* < 0.05, ** *p* < 0.01, *** *p* < 0.005, Student′s *t*-test.

Looking for a strategy aimed at improving the activity of selinexor in the poorly responsive TNBC model, we evaluated the effects of miR-34a ectopic expression on TNBC cells. miR-34a is an onco-suppressive miRNA known to be down-regulated in different tumor types [17], including TNBC [18], and previously reported to target survivin [19,20,21]. Quantitative RT-PCR results indicated that miR-34a abundance is consistently reduced in the four TNBC cell lines compared to the normal human breast cell line MCF-10A (Figure 4a). Enforced expression of miR-34a (Figure 4b) in MDA-MB-231 cells was paralleled by a time-dependent decrease of survivin mRNA expression levels (Figure 4c), and resulted in a time-dependent cell growth delay (Figure 4d). Comparable results were obtained in SUM-159 cells (Figure 4e–g).

### 2.5. miR-35a Ectopic Expression Improves the Antiproliferative and Antitumor Activity of Selinexor in TNBC Cells and Xenografts

**Figure 5:**
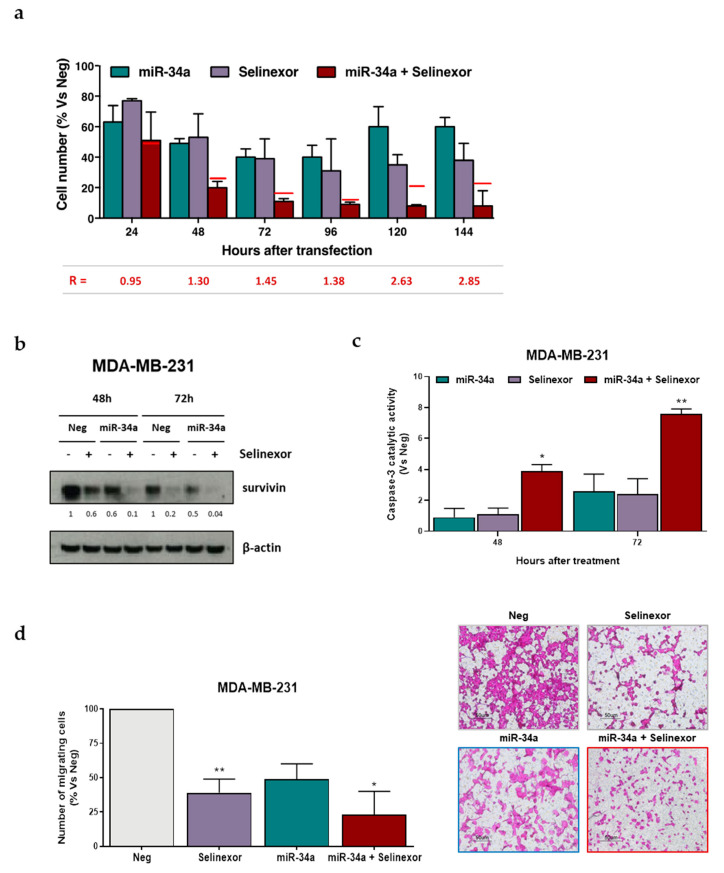
miR-34a ectopic expression improves the activity of selinexor in TNBC cells. (**a**) Effects of miR34a in combination with selinexor on MDA-MB-231 cells growth. Data are reported as percentage of growing cells with respect to Neg-transfected cells (100%), and represent mean values ± SD of three independent experiments. Red lines represent the expected additive effect of the combination, calculated as the product of the effects of the individual treatments. Synergistic ratio (R) is calculated according to [22] (R > 1, synergism; R =1, additivity; R < 1, antagonism). (**b**) Representative western blotting showing the expression of survivin in MDA-MB-231 cells after the transfection with Neg or miR-34a alone or in combination with selinexor (IC_50_ at 72 h, as reported in Table 1). β-actin was used to confirm equal protein loading on the gel. Cropped images of selected proteins are shown. For each protein, band density was quantified using ImageJ normalized to loading control and referred to respective untreated control. (**c**) Assessment of caspase-3 catalytic activity in MDA-MB-231 cells after transfection with Neg or miR-34a alone or in combination with selinexor (IC_50_ at 72 h). Data are reported as relative absorbance with respect to Neg-transfected cells, and represent the mean values ±SD of at least three independent experiments. (**d**) Left: Quantification of migration cells. Data are reported as the percentage of migrated cells with respect to Neg-transfected cells (100%), and represent mean values ± SD of three independent experiments. Right: representative photomicrographs of a transwell migration assay carried out on MDA-MB-231 cells 48 h after transfection with Neg or miR-34a either alone or in combination with selinexor. * *p* < 0.05, ** *p* < 0.01, Student′s *t*-test.

Interestingly, when miR-34a-reconstituted MDA-MB-231 cells were exposed to selinexor, it was found the miRNA synergistically cooperated with the XPO1 inhibitor to reduce cell growth (Figure 5a). The extent of the synergistic interaction increased over time, with the synergistic ratio (R) ranging from 1.3 at 48 h to 2.85 at 144 h (Figure 5a). miR-34a ectopic expression and selinexor concurred in inhibiting survivin protein expression (Figure 5b), which was accompanied by a significant time-dependent increase in caspase-3 catalytic activity, as determined in vitro by the hydrolysis of the specific synthetic substrate (Figure 5c). In addition, the combined treatment induced a greater inhibition of cell migratory capability, as detected in a migration assay, compared to that observed after miR-34a ectopic expression or selinexor exposure (Figure 5d).

**Figure 6:**
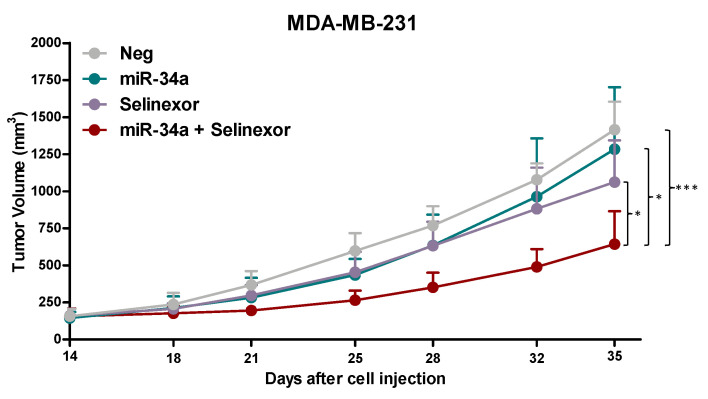
miR-34a ectopic expression improves the anti-tumor activity of oral selinexor in orthotopic TNBC xenografts. Tumor growth curves of Neg- or miR-34a-MDA-MB-231 cells orthotopically injected into SCID mice. Mice (6 mice/group) were randomly grouped to receive vehicle (ctr) or selinexor (10 mg/kg, q3-4d × 8). Each point indicates the mean tumor volume in the group ± SD. * *p* < 0.05, *** *p* < 0.005, Dunn’s *post hoc* test.

In vitro findings were then challenged in the in vivo setting by orthotopically transplanting MDA-MB-231 cells, transfected with miR-34a mimic or negative control, into SCID mice to generate xenografts. Results indicate that neither miR-34a nor selinexor as single agent appreciably affected the growth of MDA-MB-231 xenografts (Figure 6. However, consistent with in vitro data, miR-34a ectopic expression markedly improved the antitumor activity of selinexor in MDA-MB-231 xenografts, as indicated by the enhanced maximum TVI (58% versus 25% in miR-34a- and Neg-transfected cells) (Figure 6), thus confirming the positive interaction between the two treatments. Of note, the combined treatment was well tolerated, with no obvious signs of toxicity.

## 3. Discussion

In this study, we showed that selinexor is active in TNBC in vitro models, although the level of sensitivity to the drug varies among the cell lines and reflects in a different anti-tumor activity following orthotopic xenotransplantation into SCID mice. We also demonstrated that ectopic expression of miR-34a—an onco-suppressive miRNA that regulates survivin expression—significantly improved the activity of the XPO1 inhibitor in less sensitive TNBC cells and xenografts.

In spite of its documented ability to induce anti-proliferative and anti-tumor effects in TNBC experimental models [9,10,11], results from a recent Phase II trial indicated that single-agent selinexor has limited activity in heavily pretreated patients with metastatic TNBC [12]. This evidence, together with the good tolerability of selinexor, supports a try with combination regimens aimed at increasing its therapeutic effect.

Based on previous reports, it is well established that down-regulation of survivin plays a main role in the activity of selinexor in experimental models of different tumor types, including BC [10,13,14,15,16]. Here, we found that the XPO1 inhibitor impaired the intracellular distribution of survivin and induced a time-dependent depletion of the survivin cytoplasmic pool, which is responsible for the anti-apoptotic function of the protein [23]. Consistent with previous findings [24,25], we observed that selinexor reduced *survivin* gene transcription by decreasing acetylation of the STAT3 transcription factor and also induced the ubiquitination of survivin nuclear fraction, providing evidence that both mechanisms concur to the down-regulation of survivin following treatment with the XPO1 inhibitor in TNBC cells.

In the search for combination regimens able to increase the response to selinexor, we reasoned that a concomitant strategy for down-regulating survivin expression could have had a positive impact on the XPO1 inhibitor anti-tumor activity. We focused on miR-34a, an onco-suppressive miRNA shown to be down-regulated in different human tumor types [17], including TNBC [18], which was reported to negatively regulate survivin expression [19,20,21]. miR-34a is implicated in mammary epithelium homoeostasis by controlling both proliferation and fate commitment in mammary progenitors through the modulation of several pathways involved in epithelial cell plasticity and luminal-to-basal conversion [26]. Specifically, the miRNA acts as endogenous inhibitor of the Wnt/β-catenin signaling pathway thus modulating the expansion of the mammary stem cells/early progenitor pool [26]. 

It has been previously shown that miR-34a ectopic expression displays anti-tumorigenic effects in TNBC in vitro and in vivo models by silencing the proto-oncogene c-SARC [18] and by restoring the expression of the arrestin protein ARRDC3 [9]. In addition, miR-34a was reported to inhibit BC cell migration and invasion by targeting epithelial-to-mesenchymal transition-inducing transcription factors, such as TWIST1 and ZEB1 [27]. Moreover, miR-34a was shown to exert a dual suppressive effect on the FOXM1/eEF2-kinase axis, through which it negatively regulates TNBC cell proliferation, motility and invasion [28]. Interestingly, TNBC patients characterized by high miR-34a expression levels display a better survival than those showing low miR-34a levels [18,28].

Here, we found that miR-34a ectopic expression in TNBC cells down-regulated survivin, induced caspase-3 activation and inhibited in vitro cell growth and migration. Most importantly, and consistent with the evidence that miR-34a positively modulates the activity of anti-cancer drugs in experimental models of different human tumor types [29], we showed that the miRNA was able to markedly improve the anti-proliferative and anti-tumor activity of selinexor in the MDA-MB-231 TNBC model, which is poorly sensitive to the XPO1 inhibitor as single agent. In this context, we recently reported that another approach aimed at targeting apoptosis-related molecules was efficient in improving selinexor activity in TNBC. Specifically, we demonstrated that selinexor-exposed TNBC cells exhibited significantly enhanced cell growth inhibition and apoptotic rate when treated with the bi-specific antibody TRAIL-R2xCD3 [10], an antibody that targets TNF-related apoptosis-inducing ligand receptor 2 (TRAIL-R2) [30] and that already demonstrated a good ability in redirecting CD3^+^ T cells to kill TRAIL-R2-expressing TNBC cells [31].

Although both miR-34a and selinexor concur to the down-regulation of survivin protein expression, there is no doubt that other target genes of the miRNA might play a role in the enhancement of the XPO1 inhibitor effects observed in our TNBC models. 

Preclinical data generated in the present study form a solid foundation for promoting the clinical use of a miR-34a-based approach to improve the therapeutic efficacy of selinexor in TNBC patients. However, a major constraint towards translating miRNA-based strategies into human cancer therapy is related to the need for safe and efficient delivery systems. In this context, in 2013, a miR-34a mimic encapsulated in lipid nanoparticles (MRX34) entered a multicentre Phase I study in patients with advanced solid tumors showing evidence of activity in a subset of patients. However, the study was closed early due to serious immune-mediated adverse events [32]. Other miR-34a delivery systems have been proposed [17,29], and some of them have been evaluated in TNBC in vitro models, including photoresponsive gold nanoshells [33] and layer-by-layer assembled poly(lactic-co-glycolic acid) nanoparticles [34]. Both delivery approaches yielded robust suppression of miR-34a targets, which was accompanied by reduced cell proliferation. However, no in vivo data are currently available.

It is worth mentioning that, similarly to anti-cancer drugs, also miRNA-based therapeutics can activate resistance mechanisms. In this context, we previously provided evidence of a cytoprotective/resistance mechanism arising towards miR-34a reconstitution in diffuse malignant peritoneal mesothelioma cells through the persistent activation of ERK1/2 and AKT signaling [35], a well known mechanism of resistance to tyrosine receptor kinase inhibitors [36,37].

## 4. Materials and Methods

### 4.1. Cell Lines

Human TNBC cell lines (MDA-MB-231, SUM-159, HCC1937, MDA-MB-468) and the normal human breast cell line (MCF-10A) were purchased at the American Type Culture Collection (ATCC, Manassas, VA, USA). Cells were maintained in the logarithmic growth phase as a monolayer in DMEM F12 supplemented with 10% heat-inactivated fetal bovine serum, in a humidified incubator at 37 °C with a supply of 5% CO_2_/95% air atmosphere. Cell lines are tested fortnightly for the absence of Mycoplasma and periodically (every six months) monitored for DNA profile of short tandem repeats analysis by the AmpFISTR Identifiler PCR amplification kit (Thermo Fisher Scientific Inc., Waltham, MA, USA).

### 4.2. Cell Transfection 

Mimic pre-miR-34a precursor (miR-34a) and mimic negative control (Neg) were purchased as Pre-miRmiRNA precursor molecules (Thermo Fisher Scientific Inc.). MDA-MB-231 and SUM-159 cells were plated into 6-well plates at a density of 12 × 10^4^ cells/mL. Twenty-four h later, cells were transfected using Lipofectamine RNAiMAX (Thermo Fisher Scientific Inc, Waltham, MA, USA) with 20 nM of miR-34a or Neg. Cells were incubated with transfection mix for 5 h and then the transfection medium was replaced with complete medium. The expression of the miRNA in TNBC cells was checked by qRT-PCR at different timepoints after transfection.

### 4.3. Cell Growth Inhibition Assay

Selinexor (Karyopharm Therapeutics, Newton, MA, USA) was initially dissolved in DMSO, stored at −20 °C, and diluted in complete culture medium immediately before use. Control cells were exposed to 0.1% (*v*/*v*) DMSO. The anti-proliferative activity of the drug, at concentrations ranging from 0.01 to 10 µM for different intervals of exposure time, was determined by the CellTiter 96 AQueous One Solution Cell Proliferation Assay (MTS; Promega, Madison, WI, USA). The concentration able to inhibit cell growth by 50% (IC_50_) was determined graphically from the dose-response curves obtained after cell exposure to selinexor. 

To assess the effect of miR-34a ectopic expression on cell growth, TNBC cells were transfected with Neg or miR-34a as described above. At different timepoints after transfection, cells were trypsinized and counted in a particle counter (Beckman Coulter Life Sciences, Brea, CA, USA). Results were expressed as percent variation in the number of miR-34a-transfected cells compared to Neg-transfected cells. In combination experiments, at 24 h after transfection, cells were exposed to selinexor (IC_50_) or drug vehicle for 72 h. Results were expressed as percent variation in the number of treated cells compared to Neg untreated cells.

To evaluate treatment interactions, the synergistic ratio (R) was calculated as described in [38]. According to this method, the expected value of cell survival is defined as the product of the survival observed for the two treatments alone:

Expected Survival = Survival treatment A × Survival treatment B

and the R is calculated as the ratio of expected and observed survival:

R = Expected Survival/Observed Survival

where R > 1 indicates synergy while R ≤ 1 indicates the absence of synergy/additive effect.

### 4.4. Cell Cycle Distribution Analysis

Both adherent and floating cells were fixed with 70% EtOH and incubated overnight at 4 °C in staining solution containing 50 μg/mL of propidium iodide, 50 mg/mL of RNase, and 0.05% Nonidet-P40 in PBS. Samples were analyzed with a BD Accuri C6 flow cytometer (Becton Dickinson, Franklin Lakes, NJ, USA). At least 30,000 events were read, and histograms were generated using the CellQuest software according to the Modfit model (Becton Dickinson).

### 4.5. Caspase-3 Catalytic Activity 

The catalytic activity of caspase-3 was measured as the ability to cleave the specific substrate N-acetyl-Asp-Glu-Val-Asp-pNA (DEVD-pNA) by means of APOPCYTO/caspase-3 kits (Cat #K106-100; R&D System Inc, Minneapolis, MN, USA), according to manufacturer’s instructions. The hydrolysis of the substrate was monitored by spectrofluorometry with 380 nm excitation and 460-nm emission filters. Results were expressed as relative absorbance with respect to Neg-transfected cells.

### 4.6. Protein Extraction and Western Blot Analysis

Lysates were obtained from TNBC cells collected after treatment with selinexor for the indicated exposure time. Nuclear and cytosolic fractions were obtained from MDA-MB-231 and SUM-159 cells using NE-PER nuclear and cytoplasmic extraction reagents (#78835; Thermo Fisher Scientific Inc.) following the manufacturer’s protocol. Total/fractioned cellular lysates and immunoprecipitates were separated by SDS-PAGE, transferred onto nitrocellulose membranes and incubated with primary antibodies: anti-XPO1 (ab24189; Abcam, Cambridge, UK), anti-survivin (ab469; Abcam), anti-PARP-1 (#9542; Cell Signaling Technology, Danvers, MA, USA), anti-STAT3 (#4904; Cell Signaling Technology), anti-acetyl-STAT3 (#2523; Cell Signaling Technology), anti-p53 (sc-126; Santa Cruz Biotechnology, Dallas, TX, USA), and anti-FOXO3a (#2497, Cell Signaling Technology). Anti-β-actin (ab8226; Abcam), anti-HSP90 (NBP1-77944; Novus Biologicals, Centennial, CO, USA) and anti-HDAC2 (#5113; Cell Signaling Technology) antibodies were used to confirm equal protein loading on the gel, and also to show the relative purity of the cytosolic or nuclear fractions. Band density were quantified by scanning films and processing image intensities with the ImageJ 1.47v Software.

### 4.7. ELISA Assay

Survivin protein was quantified in nuclear/cytosolic cell lysate obtained from cells exposed to selinexor using Surveyor IC Human Total Survivin Immunoassay (#SUV647; R&D Systems, Minneapolis, MN, USA) according to the manufacturer’s protocol. Nuclear ubiquitinated survivin, obtained by immunoprecipitation with an anti-human survivin antibody (#ab469; Abcam) was quantified in the nuclear fraction, using CycLex Poly-Ubiquitinated Protein ELISA Kit (MBL International), according to manufacturer’s protocol. Cleaved PARP-1 were quantified using Pierce Cleaved PARP-1 Colorimetric In-Cell ELISA Kit (#62219; Thermo Fisher Scientific Inc., Waltham, MA, USA), according to the manufacturer’s protocol.

### 4.8. Quantitative RT-PCR

The expression levels of survivin mRNA were assessed by qRT-PCR using the specific TaqMan gene expression assay (Hs00153353_m1; Thermo Fisher Scientific Inc., Waltham, MA, USA) on total RNA (0.5 µg), isolated from TNBC cells using QIAzol Lysis Reagent and RNeasy Mini kit (QIAGEN, Hilden, Germany), randomly primed and reverse transcribed using the High Capacity cDNA Reverse Transcription kit (Thermo Fisher Scientific Inc.). For the evaluation of miR-34a expression levels, total RNA (1 μg) isolated using QIAzol Lysis Reagent and miRNeasy Mini Kit (QIAGEN) was reverse transcribed by miScript II RT Kit (QIAGEN). Mature miRNA expression was assayed by the specific TaqMan Assay hsa-miR-34a (TM: 000426; Thermo Fisher Scientific Inc., Waltham, MA, USA). For comparative analysis, GAPDH (PN4326317, Thermo Fisher Scientific Inc.) and RNU48 (TM: 001006; Thermo Fisher Scientific Inc.) were used as endogenous controls for genes and miRNA, respectively. Amplifications were run on the 7900HT Fast Real-Time PCR System (Thermo Fisher Scientific Inc., Waltham, MA, USA). Data were analyzed by SDS 2.2.2 software (Thermo Fisher Scientific Inc., Waltham, MA, USA) and reported as relative quantity (RQ) with respect to a calibrator sample using the 2−ΔΔCt method.

### 4.9. Migration Assay

Forty-eight h after transfection with miR-34a or Neg, TNBC cells treated or not with selinexor for 24 h were seeded in the upper chamber (1.0 × 105 cells per well) of 24-well Transwell plates equipped with polycarbonate filters (Costar; Corning Incorporated, Corning, NY, USA), in serum-free medium. MDA-MB-231 complete media were added to the lower chamber as chemoattractant. After 6 h of incubation at 37 °C, filters were fixed with 99% ethanol at −20 °C and stained with a 0.4% sulforhodamine B/1% acetic acid solution. Migrated cells were counted under an inverted microscope in five randomly chosen fields. All samples were performed in duplicate. 

### 4.10. In Vivo Experiments

All experimental protocols were approved by the institutional Ethics Committee for Animal Experimentation and the Italian Ministry of Health according to the national law (Project approval code: 1159/2015-PR). 

Orthotopic models were generated by injecting SUM-159 cells, miR-Neg- or miR 34a-MDA-MB-231 cells into the mammary fat pad of 5-week-old SCID mice. The treatment with selinexor started when tumors reached ~200 mm^3^. Selinexor was prepared as previously described [39]. Mice (6/group) were randomized to receive the drug vehicle or selinexor delivered orally (*p.o.*, 10 mg/kg) twice a week for 8 times (q3-4d × 8). Tumor growth was followed by biweekly measurements of tumor diameters with a Vernier calliper and tumor volume (TV) was calculated according to the formula: TV (mm^3^) = d^2^ × D/2, where d and D are the shortest and the longest diameter, respectively. The anti-tumor activity was assessed as TV inhibition percentage (TVI%) in treated versus control mice, calculated as follows: TVI% = 100 − (mean TV treated/mean TV control × 100). Drug treatment toxicity was determined as body weight loss and lethal toxicity. Deaths occurring in treated mice before the death of the first control mouse were ascribed to toxic effects. The origin of orthotopic xenografts was authenticated through microsatellite analysis by the AmpFISTR Identifiler PCR Amplification Kit (Thermo Fisher Scientific Inc.).

### 4.11. Statistical Analysis

Statistical analyses were performed using the GraphPad Prism software, version 4.0 (GraphPad Prism Inc., San Diego, CA, USA). Statistically significant difference between two groups was assessed by two-sided Student’s *t*-test. Statistical analyses among more than two groups were performed by two-way ANOVA with Tukey’s *post hoc* test or by Kruskall-Wallis test with Dunn’s *post hoc* test (as specified in Figure legends). A *p* value of ≤0.05 was considered statistically significant. 

## Figures and Tables

**Table 1:** Cell growth inhibitory activity of selinexor in TNBC cell lines.

Cell Line	IC_50_ (µM) ^1^		
	24 h	48 h	72 h
MDA-MB-231	>10	>10	0.05 ± 0.02
SUM-159	>10	3.1 ± 1.1	0.12 ± 0.04
HCC1937	>10	>10	1.04 ± 0.03
MDA-MB-468	3.7 ± 1.4	0.18 ± 0.06	0.03 ± 0.02

^1^ Concentration of drug required to inhibit cell growth by 50% (IC_50_), as determined after continuous exposure to selinexor for different time intervals. Data represent the mean ± SD of three independent experiments.

**Table 2:** Anti-tumor activity of selinexor in TNBC xenografts.

Xenografts Model	Treatment (mg/kg)	Schedule	Route	Max TVI% (Day) ^a^
MDA-MB-231	10	q3-4d × 8	*p.o.*	25(31)
SUM-159	10	q3-4d × 8	*p.o.*	67(28) *

^a^ Maximum tumor volume inhibition (TVI)% in treated versus control mice. In parentheses, the day on which it was assessed. * *p* < 0.01 with respect to control animals.

## Data Availability

The data presented in this study are available in the article.
